# Comparing Antibiotic Regimens for Preventing Infections After Planned Cesarean Delivery

**DOI:** 10.1097/og9.0000000000000108

**Published:** 2025-08-14

**Authors:** Daniel Gabbai, Itamar Gilboa, Anat Lavie, Yariv Yogev, Emmanuel Attali

**Affiliations:** Lis Hospital for Women's Health, Tel Aviv Sourasky Medical Center, and Faculty of Medicine, Tel Aviv University, Tel Aviv, Israel.

## Abstract

**OBJECTIVE:**

To compare the efficacy of two antibiotic regimens (cefazolin vs clindamycin plus gentamicin) for preventing infectious complications after planned cesarean delivery.

**METHODS:**

A retrospective cohort study was conducted at a tertiary university-affiliated medical center between 2012 and 2023. All women undergoing planned cesarean delivery were included and divided into two groups: The standard regimen group (control) was treated with cefazolin, and the study group, consisting of women with reported severe penicillin allergies or cephalosporin allergies, was treated with a clindamycin plus gentamicin regimen. Maternal and neonatal outcomes were analyzed with multivariate logistic regression. The primary outcome was the need for inpatient antibiotic treatment; the secondary outcome was readmission for obstetric or gynecologic complications.

**RESULTS:**

During the study period, 145,883 deliveries occurred in our department, of which 17,693 (12.1%) were by planned cesarean deliveries. Among these, 10,588 women (94.1%) received cefazolin, and 658 women (5.9%) received clindamycin plus gentamicin. Infectious complications were less frequent in the control group. The need for inpatient antibiotic treatment was 5.9% in the cefazolin group compared with 15.2% in the clindamycin plus gentamicin group (*P*<.001), and readmission rates were 1.8% vs 3.8%, respectively (*P*=.001). On multivariate analysis, the alternative regimen group had significantly higher odds of requiring inpatient antibiotics after cesarean delivery (adjusted odds ratio [aOR] 2.1, 95% CI, 1.54–2.80, *P*<.001) and readmission (aOR 1.95, 95% CI, 1.19–3.18, *P*=.008).

**CONCLUSION:**

Cefazolin may be more effective than clindamycin plus gentamicin in preventing infectious complications after planned cesarean delivery. This study emphasizes the importance of careful assessment of β-lactam allergies to guide optimal antibiotic choices. For women allergic to standard regimens, alternative strategies should be considered to reduce postoperative infections and complications.

## Abstract 2

Cefazolin may be more effective than clindamycin plus gentamicin for infection prevention after elective cesarean delivery, underscoring the need for careful β-lactam allergy assessment.

The rate of cesarean deliveries has increased over the past two decades, with an estimated 29.7 million procedures performed annually worldwide.^1^ This rise is associated with increased maternal morbidity and mortality, as well as extended postpartum hospitalizations and higher readmission rates.^2^ Consequently, cesarean delivery has emerged as a significant public health concern with both medical and economic implications.^3^

Because cesarean delivery is generally classified as a clean-contaminated procedure, antibiotic prophylaxis is recommended to reduce the risk of puerperal infections, including both endometritis and surgical site infections.^4^

The American College of Obstetricians and Gynecologists (ACOG) recommends antibiotic prophylaxis for all cesarean deliveries, ideally administered within 60 minutes before surgery begins.^5^ Studies have demonstrated that prophylactic antibiotics significantly reduce the risk of febrile morbidity, wound complications, and endometritis associated with cesarean delivery.^6^ The preferred antibiotic is a first-generation cephalosporin; however, for patients allergic to β-lactam agents, a single dose of clindamycin combined with an aminoglycoside is a reasonable alternative. Nonetheless, ACOG has noted that this recommendation is based on limited available data.^5^

Although previous studies have investigated outcomes by comparing women treated with cephalosporins with those given alternative agents,^7^ our study sought to minimize confounding factors by focusing on women undergoing planned cesarean deliveries.

## METHODS

This retrospective cohort study included all women who underwent planned cesarean delivery at a university-affiliated tertiary medical center between 2012 and 2023. To reduce confounding, only planned cesarean deliveries were included; we excluded those performed intrapartum or urgently. In addition, women without documented prophylactic antibiotic data were excluded. Participants were categorized according to their prophylactic regimen: The standard regimen group (control) was treated with cefazolin, and the study group, comprising women with severe penicillin or cephalosporin allergies, was treated with clindamycin plus gentamicin. A comparative analysis of demographic and clinical characteristics was conducted between the standard and alternative regimen groups.

At our university-affiliated hospital, cesarean deliveries are performed with a modified Misgav–Ladach technique.^8^ Prophylaxis antibiotics are given on the basis of patients' allergies within 30 minutes before the incision.^9^ The standard regimen is a 2-g dose of cefazolin; however, a 3-g dose is considered for women weighing 120 kg or more. The alternative regimen antibiotics are clindamycin (600 mg) and gentamycin (5 mg/kg).^10^

The surgical approach involves a straight transverse incision, positioned slightly higher than the traditional Pfannenstiel incision. Subcutaneous tissue is left intact except at the midline, and the rectus sheath is divided along its fibers. The rectus muscles are separated by blunt dissection rather than cutting, and the peritoneum is opened by gently stretching with the index fingers to avoid the use of sharp instruments. The uterus is opened with an index finger and manually enlarged between the thumb and index finger of each hand.

For closure, the uterus is sutured with a double-layer technique with polyglactin 910 (Vicryl) or Spiral Monocryl (Stratafix) sutures at the discretion of the surgeon. The abdominal fascia is closed continuously with polyglactin 910 (Vicryl) or polydioxanone, and the subcutaneous layer is closed if it exceeds 2 cm in depth. Skin closure is achieved through subcuticular sutures, staples, or absorbable staples.^10^

The primary outcome was the requirement for inpatient antibiotic therapy, reflecting the presence of infectious complications necessitating treatment during the index hospitalization for cesarean delivery. Secondary outcomes included readmission for obstetric complications such as surgical site infections, endometritis, pelvic abscess, late postpartum hemorrhage, postpartum preeclampsia, and retained placental products, as well as other related obstetric or gynecologic complications. Additional secondary outcomes during the index hospitalization included red blood cell (RBC) transfusion and length of hospital stay. Neonatal outcomes included Apgar score lower than 5 and umbilical artery pH less than 7.2.

Medical records were obtained from the computerized delivery room logbooks, providing a comprehensive set of demographics, obstetric, and clinical characteristics. These included maternal age, pregestational body mass index (BMI, calculated as weight in kilograms divided by height in meters squared), parity, gravidity, history of cesarean deliveries, gestational age at delivery, multiple pregnancies, and fertilization method. Additional data on pre-existing or gestational conditions such as preeclampsia and diabetes mellitus were also extracted. Surgical variables such as operative time and skin closure technique (nonabsorbable staples, absorbable staples, or absorbable sutures) were additionally recorded.

*Leukocytosis* was defined as a leukocyte count above 16,000/microliter, anemia as hemoglobin levels below 11 g/dL, and thrombocytopenia as a platelet count below 150,000/microliter. *Prolonged hospitalization* was defined as a length of stay exceeding 5 days. *Readmission* was defined as any admission to the gynecologic department for obstetric complications related to the surgery within 45 days after the procedure.

Univariate analyses were performed to assess differences between groups. Continuous variables were analyzed with two-tailed unpaired Student *t* tests or Mann–Whitney *U* tests as appropriate according to data distribution. Categorical variables were compared with χ^2^ or Fisher exact tests. A multivariable logistic regression model was then constructed to evaluate the association between independent variables and outcomes, with adjustment for covariates that were statistically significant in the univariate analysis.

Data analysis was performed with SPSS 21.0. *P*<.05 was considered statistically significant. All analyses used anonymized data; therefore, informed consent was not required. The study was approved by the Tel Aviv Sourasky Medical Center IRB (No. TLV-0284-08, July 10, 2024).

## RESULTS

**Fig. 1.**
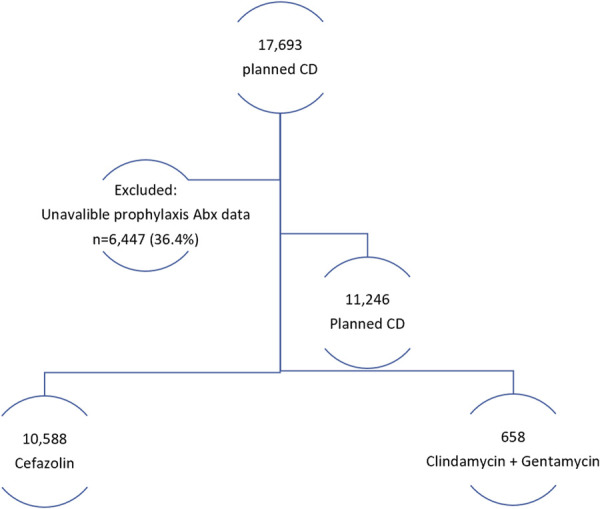
Study population. CD, cesarean delivery.

During the study period, a total of 145,883 deliveries occurred at our center, with 17,693 cesarean deliveries being planned. After the exclusion criteria were applied, 11,246 women were eligible for analysis (Fig. 1). Of these, 10,588 (94.1%) received cefazolin, and 658 (5.9%) were administered clindamycin and gentamicin.

*Gabbai. Antibiotics for Infection Prevention in Cesarean. O&G Open 2025*.

Table 1 outlines the indications for cesarean delivery. The most common indication was a history of two or more cesarean deliveries or other uterine scarring, followed by malpresentation, a single previous cesarean delivery with patient request, multiple pregnancy, patient request for cesarean delivery, suspected macrosomia, abnormal placentation, previous obstetric anal sphincter injury, prior shoulder dystocia, cephalopelvic disproportion, and preeclampsia.

**Table 1.** Cesarean Delivery Indications

Indication	Prevalence (%)
Previous cesarean deliveries (more than 2) or other myometrium scar	33.1
Malpresentation	18.9
Previous cesarean delivery and patient request	13.3
Multiple pregnancy	9.3
Patient request for cesarean delivery	7.3
Other elective indications	7.3
Macrosomia suspected	6.1
Abnormal placentation	2.6
Previous obstetric anal sphincter injury	1
Previous shoulder dystocia	0.6
Cephalopelvic disproportion	0.3
Preeclampsia toxemia	0.3

Table 2 details the demographic and obstetric characteristics of both groups. Women in the alternative regimen group were older (mean±SD age 36.1±5.45 years vs 35.1±5.24 years, *P*<.001) and had higher BMI (23.7 [interquartile range 20.9–27.5] vs 23.1 [interquartile range 20.6–26.8], *P*=.014). They also had a higher prevalence of assisted reproductive technology use (28.2% vs 23.2%, *P*=.007), greater use of general anesthesia (5.1% vs 2%, *P*<.001), and a higher incidence of preeclampsia (7.6% vs 4.6%, *P*<.001). Median surgery time was also longer in the alternative regimen group (65 minutes [interquartile range 48–72 minutes] vs 59 minutes [interquartile range 46–68 minutes]). In addition, women in this group were more likely to have nonabsorbable staples used for skin closure (36.6% vs 31.5%, respectively). No significant differences were found in leukocytosis, anemia, or thrombocytopenia between the groups based on complete blood count parameters. In addition, there were no significant differences in neonatal outcomes, including 5-minute Apgar score lower than 7 and umbilical artery cord pH below 7.2.

**Table 2.** Comparison of Demographic and Obstetric Characteristics and Maternal and Neonatal Outcomes

Factor	Standard Regimen (Cefazolin) (n=10,588)	Alternative Regimen (Clindamycin+Gentamicin) (n=658)	*P*
Maternal age (y)	35.17±5.24	36.1±5.45	<.001
Gestational week	38.3 (37.5–39)	38.2 (37.2–39)	.002
BMI (kg/m^2^)	23.1 (20.6–26.8)	23.7 (20.9–27.5)	.01
Nulliparity	3,475 (33.1)	227 (34.8)	.36
Previous cesarean delivery	4,951 (47.1)	300 (46)	.59
Assisted reproductive technology	2,133 (23.2)	165 (28.2)	.007
Multiple pregnancy	1,349 (12.8)	92 (14.2)	.34
Preeclampsia	485 (4.6)	50 (7.6)	<.001
Gestational diabetes	2,195 (20.7)	155 (23.6)	.08
Pregestational diabetes	219 (2.1)	19 (2.9)	.16
General anesthesia	198 (2)	32 (5.1)	<.001
Preoperative anemia	61 (25.5)	13 (41.9)	.08
Preoperative leukocytosis	185 (1.9)	17 (3)	.09
Preoperative thrombocytopenia	1,737 (18.3)	104 (18.2)	.99
Surgery time (min)	59 (46–68)	65 (48–72)	<.001
Skin closure method			
Nonabsorbable staples	3,042 (31.5)	219 (36.6)	.02
Absorbable subcutaneous staples	5,894 (61.1)	349 (58)	
Absorbable subcutaneous sutures	712 (7.4)	34 (5.6)	
Neonatal outcomes			
5-min Apgar score lower than 7	49 (0.5)	5 (0.8)	.25
Umbilical artery pH below 7.2	318 (6.8)	26 (9.6)	.08
Pregnancy outcomes			
Antibiotic use after cesarean delivery	620 (5.9)	100 (15.2)	<.001
Length of stay longer than 5 d	763 (7.2)	77 (11.7)	<.001
Packed cell transfusion,	174 (1.6)	28 (4.3)	<.001
Readmission for obstetric or gynecologic complications	194 (1.8)	25 (3.8)	.001

BMI, body mass index.

Data are mean±SD, median (interquartile range), or n (%) unless otherwise specified.

**Fig. 2.**
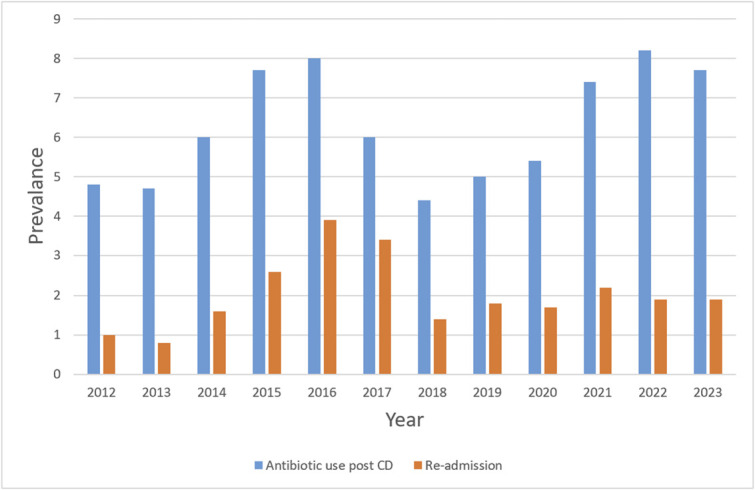
Annual prevalence of pregnancy outcomes after cesarean delivery (CD).

The overall prevalence of adverse outcomes was as follows: 6.4% for inpatient antibiotic use after cesarean delivery, 1.9% for readmission for obstetric or gynecologic complications, 7.5% for prolonged hospitalization (more than 5 days), and 1.8% for packed RBC transfusion. Figure 2 presents trends in the annual prevalence of two representative outcomes. Women who received the alternative antibiotic regimen had significantly higher rates of antibiotic use after cesarean delivery (15.2% vs 5.9%, *P*<.001), prolonged hospitalization (11.7% vs 7.2%, *P*<.001), packed RBC transfusion (4.3% vs 1.6%, *P*<.001), and readmission (3.8% vs 1.8%, *P*=.001).

*Gabbai. Antibiotics for Infection Prevention in Cesarean. O&G Open 2025*.

In a multivariable logistic regression model (Table 3) controlling for maternal age, gestational week, BMI, use of assisted reproductive technology, general anesthesia, preeclampsia, skin closure method, and surgery time, the alternative prophylactic antibiotic regimen emerged as an independent risk factor for each outcome separately. Specifically, it was associated with higher odds of postcesarean antibiotic use (adjusted odds ratio [aOR] 2.1, 95% CI, 1.54–2.80, *P*<.001), readmission for obstetric or gynecologic complications (aOR 1.95, 95% CI, 1.19–3.18, *P*=.008), and packed RBC transfusion (aOR 1.98, 95% CI, 1.12–3.52, *P*=.02).

**Table 3.** Multivariate Logistic Regression: Alternative Regimen Compared With Standard Regimen for Delivery Outcomes

Outcome	cOR (95% CI)	aOR (95% CI)*	*P*
Primary			
Antibiotic use after cesarean delivery	2.8 (2.29–3.62)	2.1 (1.54–2.80)	<.001
Secondary			
Packed cell transfusion	2.66 (1.77–3.99)	1.98 (1.12–3.52)	.02
Readmission for obstetric or gynecologic complications	2.16 (1.38–2.23)	1.95 (1.19–3.18)	.008
LOS longer than 4 d	1.71 (1.33–2.2)	1.25 (0.89–1.76)	.19

cOR, crude odd ratio; aOR, adjusted odd ratio; LOS, length of stay.

* The analysis was controlled for maternal age, gestational week, body mass index, use of assisted reproductive technology, general anesthesia, preeclampsia, skin closure method, and surgery time.

## DISCUSSION

Our study aimed to assess infectious complications in women undergoing planned cesarean delivery, comparing outcomes between those who received cefazolin as standard antibiotic prophylaxis and those given clindamycin plus gentamicin because of penicillin or cephalosporin allergies. The alternative antibiotic regimen (clindamycin plus gentamicin) was found to be an independent risk factor for adverse outcomes, including the primary outcome of postcesarean antibiotic use and the secondary outcome of readmission for obstetric or gynecologic complications.

The prevalence of planned cesarean delivery varies by region and health care setting. In the United States, approximately 13.8% of deliveries are planned cesareans^11,12^; some studies from other countries report a prevalence of 18.7%.^13^ In our study, the prevalence of planned cesarean delivery was 12.1%, which is consistent with previous reports.

According to ACOG, prophylactic antibiotics are recommended for all women undergoing cesarean delivery, with first-generation cephalosporins being the preferred choice unless significant drug allergy reactions such as anaphylaxis, angioedema, respiratory distress, or urticaria are reported.^5^ In cases of significant β-lactam allergy, ACOG suggests using a combination of clindamycin and an aminoglycoside as a reasonable alternative. However, there are limited efficacy data comparing these alternative antibiotics with cefazolin. Moreover, several studies have shown that adding a macrolide antibiotic such as azithromycin^14^ to the standard regimen improves outcomes, although this benefit has been demonstrated mainly in unscheduled cesarean delivery,^15,16^ which was excluded from our study.

In this study, we investigated obstetric complications, particularly infectious outcomes, between two antibiotic regimen groups after planned cesarean delivery. The primary outcome was the requirement for inpatient antibiotics after delivery, and the secondary outcome was readmission for any obstetric complication within 45 days, both serving as indirect indicators of infectious complications. The most common infections occurring in the postoperative period after cesarean delivery, as well as those leading to readmission, include surgical site infection,^17^ endometritis,^18,19^ and urinary tract infections.^20^ Notably, the primary and secondary outcomes often stem from similar underlying causes such as infection or surgical complications, highlighting the interconnected nature of these clinical issues.

Surgical site infections after cesarean delivery are typically polymicrobial,^21^ involving both gram-positive and gram-negative organisms such as *Staphylococcus aureus*, coagulase-negative staphylococci, *Escherichia coli*, and *Enterococcus* species, among others.^22,23^ These pathogens reflect the endogenous flora of the skin and lower genital tract, as well as potential contamination from the surgical environment. The increased rates of primary and secondary outcomes observed in the alternative regimen group may be attributed to the effectiveness of cefazolin in controlling skin flora compared with non–β-lactam agents.^24^

Our findings are consistent with those of a recent study^7^ comparing standard and alternative prophylactic antibiotic regimens administered before cesarean delivery that found that the use of noncefazolin antibiotics was associated with increased odds of cellulitis and endometritis. However, that study included all types of cesarean deliveries, regardless of the urgency of the procedure, a factor known to influence outcomes.^25^ In contrast, our study exclusively focused on women undergoing planned cesarean deliveries. Although the broad inclusion criteria of the previous study provide valuable generalizability, they also introduce variability that may confound the relationship between antibiotic regimen and infection outcomes. By narrowing our focus, our study isolates this effect in a more controlled setting, offering insights that are directly applicable to planned cesarean deliveries.

Previous research has investigated risk factors for adverse obstetric outcomes in women undergoing cesarean delivery. Surgical factors, including operative time and skin closure method,^26^ have been shown to influence these outcomes. In our cohort, although differences in surgical variables were observed in the univariate analysis, they did not alter the association between the antibiotic prophylaxis regimen and the outcomes in the multivariate model. In addition, prepartum anemia is a known risk factor for both infectious complications and the need for RBC transfusion,^27^ and although its prevalence did not differ significantly between study groups, we conducted a subanalysis adjusted for anemia. The association between the antibiotic regimen and outcomes remained independent in this analysis.

Several studies have identified maternal infection as a significant risk factor for the need for RBC transfusion after cesarean delivery. For instance, Kvalvik et al^28^ reported that women with surgical site infections were five times more likely to require a blood transfusion (odds ratio 5.1, 95% CI, 1.4–18.8). In our study, RBC transfusion was included as an outcome rather than a predictor based on its known association with infectious and hemorrhagic complications. Given that we evaluated indirect markers of infection, we considered transfusion to be a clinically meaningful and measurable consequence that may reflect underlying infectious morbidity. This observed association between maternal infection and increased transfusion rates may help explain our findings in that the alternative regimen group demonstrated a higher incidence of infectious complications. However, we acknowledge that this is an observational study, and the apparent association between the antibiotic regimen and transfusion rates is hypothesis generating and should be interpreted with caution. Further prospective studies are needed to determine causality.

In addition to the factors mentioned above, the efficacy of these two regimens may be explained by their mechanisms of action and effects on bacterial cells. Cephalosporins are generally bactericidal, working by inhibiting bacterial cell wall synthesis, which leads to cell lysis and death. This makes them effective against a broad range of gram-positive cocci and many gram-negative bacilli, making them suitable for treating various infections.^29^ Although gentamicin exerts bactericidal activity, clindamycin is primarily bacteriostatic. Clindamycin inhibits bacterial protein synthesis by binding to the 23S RNA of the 50S ribosomal subunit, preventing bacterial growth and replication. Under certain conditions and at higher concentrations, clindamycin can exhibit bactericidal activity against specific pathogens.^30^ This combination provides coverage through different mechanisms; however, the bacteriostatic nature of clindamycin may result in less effective eradication of certain pathogens compared with bactericidal agents. Consequently, this incomplete coverage might contribute to the differences observed in clinical outcomes between the antibiotic regimens.

The clinical implications of our study highlight the importance of selecting appropriate antibiotic prophylaxis for planned cesarean deliveries, especially in patients with cephalosporin allergies. Shenoy et al^31^ advocated for the involvement of all health care professionals, beyond allergists, in assessing reported penicillin allergies before penicillin or other β-lactam antibiotics are excluded from treatment options. It remains unclear whether this approach should be applied to obstetric patients. Our findings indicate that the alternative regimen of clindamycin plus gentamicin, although necessary for those with β-lactam allergies, is associated with increased risks of infectious complications, prolonged hospital stays, and readmission. This underscores the need for thorough allergy assessment before cesarean delivery to confirm true β-lactam allergies because only 10–20% of patients reporting a history allergy are truly allergic,^32^ potentially allowing more patients to receive cefazolin, which demonstrated a lower complication rate. For patients requiring alternative regimens, enhanced monitoring and preventive strategies, for instance, multiple doses of the alternative regimen, should be considered to minimize the risk of postoperative infections and other adverse outcomes. By identifying these risks and emphasizing careful antibiotic selection, this study informs practices aimed at improving maternal outcomes and optimizing resource use in obstetric care.

This study, while revealing the superiority of cefazolin over clindamycin plus gentamicin in preventing infectious complications after planned cesarean delivery, is limited by its retrospective design. Future prospective multicenter trials are needed to validate these findings and to assess the long-term maternal and neonatal outcomes. In addition, further research is required to evaluate alternative antibiotic regimens for patients with β-lactam allergies, particularly personalized approaches based on allergy profiles and microbiologic data. Exploring the cost-effectiveness and development of standardized guidelines for prophylactic antibiotic use in this population would also be valuable.

A major strength of our study is its large sample size, which provides robust statistical power and enhances the generalizability of the findings across similar patient populations. In addition, by focusing exclusively on planned cesarean delivery, we controlled for potential confounding factors related to emergency or intrapartum cesarean delivery, allowing more precise evaluation of prophylactic antibiotic efficacy in a controlled setting. Another strength is our use of multivariable logistic regression, which adjusts for several key demographic and clinical factors, thereby offering a more accurate estimation of the independent association between the antibiotic prophylaxis regimen and clinical outcomes.

However, our study has limitations. First, the outcomes reflect indirect indicators of infectious complications rather than direct diagnoses of endometritis or surgical site infections. In addition, as a retrospective cohort study, it is susceptible to selection bias, particularly as a result of missing data on prophylactic antibiotic use in some patients (although these patients were excluded from the analysis). This may introduce potential inaccuracies in the recorded data, which could affect the reliability of the outcome measures. Although we controlled for major confounders, unmeasured factors may still contribute to the observed differences between the antibiotic groups. Furthermore, we were unable to verify the surgical team's adherence to the protocol mentioned previously, including the timing of prophylactic antibiotic administration, which may have influenced our results. Information on the identity of the operating surgeon was not available in our dataset and therefore could not be included in the analysis. Furthermore, data on prenatal antibiotic use were not consistently available and thus could not be assessed, although such exposure may alter maternal flora and influence infectious outcomes. Finally, the potential for temporal variations in cesarean delivery practices, antibiotic protocols, and data reporting over the 12-year period, coupled with the study being conducted at a single tertiary care center, may limit its external validity and affect the generalizability of the findings to other institutions with different patient populations and care practices.

In conclusion, our study found an association between cefazolin and a lower incidence of infectious complications compared with clindamycin plus gentamicin after planned cesarean delivery. Women receiving the alternative regimen because of cephalosporin allergies had higher rates of postcesarean antibiotic use and readmissions. These findings emphasize the importance of thorough allergy assessments and suggest the need for alternative strategies to improve outcomes in this population. However, because this is an observational study, further randomized controlled trials are necessary to assess the effectiveness and cost-effectiveness of second-line antibiotics for prophylaxis in mothers who cannot receive first-line agents.
